# Expression Profiles of Exosomal miRNAs in Gaucher Patients and Their Association With Severity of Bone Involvement

**DOI:** 10.1002/jimd.70061

**Published:** 2025-07-06

**Authors:** Irene Serrano‐Gonzalo, Laura López de Frutos, Maria Sancho‐Albero, Mercedes Roca‐Espiau, Ralf Köhler, Pilar Giraldo

**Affiliations:** ^1^ Fundación Española Para el Estudio y Tratamiento de la Enfermedad de Gaucher y Otras Lisosomales (FEETEG) Zaragoza Spain; ^2^ Department of Biochemistry and Molecular Biology University of Zaragoza Zaragoza Spain; ^3^ Gaucher Disease Research Group (GIIS‐012) Instituto de Investigación Sanitaria de Aragón Zaragoza Spain; ^4^ Grupo de Investigación Mecanismos de Enfermedad Crónica e Investigación Traslacional (MECIT) Zaragoza Spain; ^5^ Instituto de Investigación Sanitaria de Aragón (IIS Aragón) Zaragoza Spain; ^6^ Instituto de Nanociencia y Materiales de Aragón (INMA), CSIC‐Universidad de Zaragoza Zaragoza Spain; ^7^ Networking Research Center in Biomaterials, Bioengineering and Nanomedicine (CIBERBBN) Instituto de Salud Carlos III Madrid Spain; ^8^ Department of Chemical and Environmental Engineering University of Zaragoza Zaragoza Spain; ^9^ Fundación Agencia Aragonesa Para la Investigación y el Desarrollo (ARAID) Zaragoza Spain; ^10^ Servicio de Hematología Hospital QuirónSalud Zaragoza Spain

**Keywords:** biomarkers, bone disease, exosomes, Gaucher disease, miRNA

## Abstract

Bone manifestations are one of the most prevalent complications in patients with Gaucher disease (GD). Bone involvement is evaluated by using imaging methods, and there are different scores to assess its severity. However, there are no biomarkers that allow us to predict these manifestations. In recent years, several miRNAs have been associated with bone involvement and postulated as excellent bioavailable biomarkers. This study aims to identify a miRNA expression profile from plasma exosomes and to associate it with the severity of bone involvement in patients with GD. This study included 60 untreated patients with GD with bone involvement, who were classified according to the S‐MRI score into three groups: mild disease (MiBD; S‐MRI < 5), moderate disease (MoBD; S‐MRI: 5–11), or severe disease (SBD; S‐MRI > 11). Plasma exosomes were purified, and miRNAs were extracted and identified by next‐generation sequencing (NGS) technology. Differentially expressed miRNAs were validated by droplet digital PCR (ddPCR). In the patients' groups classified by S‐MRI, the median ages (Q1–Q3) were: MiBD 19.0 (4.00–40.00), MoBD 40.5 (28.25–56.00), and SBD 37.5 (31.25–47.00) years. When comparing groups, we found 12 differentially expressed exosomal miRNAs. After validation, four miRNAs were identified as differentially expressed: hsa‐miR‐127‐3p, hsa‐miR‐184, hsa‐miR‐197‐3p, and hsa‐miR‐660‐5p. Notably, hsa‐miR‐127‐3p, hsa‐miR‐660‐5p, and hsa‐miR‐184 were correlated with the presence of infarcts, necrosis, and the degree of infiltration into the spine, pelvis, and femur. These three miRNAs could serve as bioavailable biomarkers to assess bone disease in GD, and further revalidation with a higher number of patients.

## 1 Introduction

Gaucher disease (GD) (OMIM#230800 for type 1, OMIM#230900 for type 2, and OMIM#231000 for type 3) is a lysosomal storage disease with a low incidence (1:50 000‐100 000) and autosomal recessive inheritance [1]. This inborn error of glycolipid metabolism is caused by the presence of pathogenic variants in the *GBA1* gene (MIM*606463), which encodes the lysosomal acid hydrolase β‐acid glucosidase or glucocerebrosidase (GCase, EC 3.2.1.45). The main clinical manifestations are linked to abnormal deposits such as hepatosplenomegaly, bone marrow infiltration, secondary cytopenias, and skeletal complications. Less frequently, patients with GD also exhibit pulmonary pathology and neurological involvement [2, 3].

Based on the severity of neurological involvement, patients are classified into three types: type 1, known as the non‐neuronopathic form; type 2 or acute neuronopathic form; and type 3, which presents an intermediate phenotype (chronic neuronopathic form). Despite the continued use of this classification, it is recognized that clinical symptoms can vary widely among patients, with overlaps between the groups. This classification is based on neurological manifestations and does not account for other symptoms, such as bone disease, that patients with GD frequently experience [2, 3].

Bone involvement is highly prevalent among patients diagnosed with GD, being estimated to be present in up to 94% of adults and 81% of children [4, 5]. The most common manifestations vary depending on age at diagnosis, with bone marrow infiltration (82% in adults vs. 38% in children), Erlenmeyer flask deformity (60% in adults vs. 49% in children), and acute pain in long bones (bone crises in 40% of adults) being some of the most frequent presentations [4, 5]. Complications such as necrosis, infarcts, or the previously mentioned bone crises, result from intraosseous vascular obstructions, which can significantly impact patients' quality of life and, in some cases, lead to disability [6].

In addition to bone marrow infiltration and intraosseous vascular phenomena, these patients frequently exhibit reduced bone mineral density, manifesting as osteopenia or osteoporosis. While the pathophysiology of these bone manifestations is not yet fully understood, several factors, including overexpression of various cytokines, altered bone vascularity, and the accumulation of Gaucher cells, play a significant role in their development. Splenectomy has also been recognized as an important factor, with splenectomized patients experiencing increased chronic inflammation and more severe bone involvement [6].

Regarding biomarkers, the most commonly used biomarkers for diagnosis and monitoring in GD are not directly associated with bone disease, but instead reflect the primary storage material (glucosylsphingosine) and the inflammatory component (CCL18/PARC cytokine and chitotriosidase activity) [7, 8]. Similarly, markers of bone remodeling or pro‐inflammatory cytokines do not correlate with the severity of bone involvement [9, 10]. However, recent research utilizing artificial intelligence has identified potential biomarkers that may aid in the early detection of skeletal alterations in patients with GD1 [11].

Micro‐RNAs (miRNA) are short RNA sequences (< 30 nucleotides) that are not translated into protein but act as regulators of gene expression by inhibiting the translation of mature mRNA [12]. Since the early 2000s, several studies have demonstrated the importance of this regulatory mechanism in bone maintenance, identifying numerous miRNAs that regulate osteoclast and osteoblast activity [13, 14, 15]. Furthermore, a link between miRNAs and the pathogenesis of GD has been established, with miR‐127‐5p identified as a regulator of the GCase transporter [16].

More than 30 miRNAs have been identified as regulators of genes involved in osteoclasts and osteoblasts differentiation [14, 15]. The potential of analyzing miRNA expression profiles as biomarkers for osteoporosis and for assessing the risk of bone fractures has already been proposed [14, 15]. However, while miRNA associated with GD have been studied, a profile defining the relationship between these small molecules and the severity of bone involvement in GD has not been established [17, 18, 19].

Over the last decade, exosomes have emerged as promising diagnostic markers for a variety of diseases, offering new opportunities for identifying novel biomarkers and therapeutic targets [20]. Exosomes are nano‐sized extracellular vesicles (EVs) measuring 50–150 nm in diameter, with a lipid bi‐layer, originating from the endocytic pathway. They are secreted by nearly all cell types of the organism, making them key elements in intercellular communication processes [21, 22]. This role is mediated through the transport and protection of specific cellular cargoes (i.e., RNAs content), which reflect the conditions of the parental cells [21, 23]. These properties make exosomes ideal candidates as diagnostic markers for various diseases, including lysosomal disorders [24, 25]. In fact, in 2020, evidence was presented that EVs could serve as biomarkers in GD patients, showing that decreased GCase activity influences the pool of secreted EVs [26].

The main objective of this study is to identify different exosomal miRNA expression patterns associated with the severity of bone involvement in patients with GD. To the best of our knowledge, this is the first study to examine whether miRNAs present in blood exosomes from patients with GD can serve as biomarkers for predicting the severity of bone disease.

## 2 Material and Methods

### 2.1 Patients

This study included untreated patients with GD from the Gaucher Spanish Registry, created by the Spanish Foundation for the Study and Therapeutics of Gaucher and other Lysosomal Diseases (FEETEG). Demographic, clinical, and imaging data (magnetic resonance image) were collected, and plasma samples were utilized for this study. Samples were provided by the Biobank of the Aragon Health System, integrated in the Spanish National Biobanks Network (PT20/00112), and they were processed following standard operating procedures with the appropriate approval of the Ethics and Scientific Committees. All participants provided written informed consent, and the study received approval from the Research Ethics Committee of the Community of Aragon (PI20/264).

Patients were classified into three groups according to the severity of their bone involvement, as determined by the Spanish magnetic resonance image (S‐MRI) score [27]. The range of S‐MRI scores for each group was defined based on previously published equivalences [28]: scores below 5 for the mild bone disease group (MiBD), scores between 5 and 11 for the moderate bone disease group (MoBD), and scores above 11 for the severe bone disease group (SBD).

The registry contains over 416 patients, allowing us to conduct a selection of cases based on the following inclusion and exclusion criteria.

#### 2.1.1 Inclusion Criteria

- Patients diagnosed with GD by enzymatic analysis and identification of biallelic variants in the *GBA1* gene.
- Plasma sample volume equal to or greater than 1.5 mL, stored in the Biobank of Aragon, at diagnosis or prior to start of the therapy.
- Available demographic, clinical, analytical, and imaging data (MRI) from the same date as the plasma sample.

#### 2.1.2 Exclusion Criteria

- Patient with GD under treatment, enzyme replacement therapy (ERT), or substrate reduction therapy (SRT).
- Patients with a plasma volume of less than 1.5 mL.
- Imaging tests (MRI) not physically available for review.
- Lack of informed consent to use the data for research.

### 2.2 Exosome Isolation and miRNA Extraction

The purification of total miRNA from exosomes present in plasma was performed in two steps, following the manufacturer's protocol for exosome extraction. Exosomes were isolated from plasma using the miRCURY Exosome Serum/Plasma kit (QIAGEN, Hilden, Germany) and miRNA was extracted from exosomes using the miRNeasy Serum/Plasma Advance kit (QIAGEN, Hilden, Germany). The quantification was performed by spectrophotometry using a NanoDrop One/OneC (Thermo Fisher Scientific, Waltham, MA, USA).

### 2.3 Exosomes Characterization

For exosome characterization, 10 samples were randomly selected: three from the MiBD group, three from the MoBD group, and four from the SBD group. Additionally, three samples from healthy controls, with negative enzymatic and genetic results, matched by age and sex, were included for characterization. Transmission electron microscopy (TEM) was performed using a Tecnai T20 transmission electron microscope (ThermoFisher, USA) operated at 200 kV in the Advanced Microscopy Laboratory (LMA) at the University of Zaragoza. Samples were prepared by drop‐casting 20 μL of the exosome suspension onto a Formvar carbon TEM grid. Fixation was first performed with 2% paraformaldehyde in PBS, followed by post‐fixation with 2.5% glutaraldehyde in water. Finally, staining was done with 2% uranyl acetate in water.

Nanoparticle tracking analysis (NTA) (Nanosight NS200, Malvern Panalytical) was used to evaluate the hydrodynamic diameter and concentration (particles/mL) of the exosomes isolated from patients with GD. Zeta potential (surface charge) was determined at pH 7 in PBS (Brookhaven 90 plus and ZetaPALS Software). A Pierce BCA protein assay (ThermoFisher, USA) was performed to estimate the total protein content of the exosome samples. Finally, to verify that the isolated vesicles were exosomes rather than other EVs (e.g., ectosomes, microvesicles, apoptotic bodies), a MACSPlex EV Kit IO (Miltenyi Biotec, Germany) was used, following manufacturer instructions, to quantify the expression of exosomal proteins by Flow Cytometer Gallios (Beckman Coulter, USA).

### 2.4 Next‐Generation Sequencing (NGS)

NGS technology was performed by bioNova Científica S.L. (Madrid, Spain). Libraries were prepared using the QIAseq miRNA Library Kit (QIAGEN), with 5 μL of RNA converted into miRNA NGS libraries. Library preparation quality was assessed using capillary electrophoresis (Tape D1000) and the library was sequenced on a NextSeq (Illumina Inc.) sequencing instrument according to the manufacturer's instructions.

Primary analysis was carried out using CLC Genomics Server 21.0.4, employing the “QIAseq miRNA Quantification” workflow with default parameters to map the reads to miRBase version 22. Version 22 contains 38 589 entries representing miRNA hairpin precursors and 48 885 mature miRNA products across 271 species. TMM (trimmed mean of *M*‐values) normalization was applied to calculate effective library sizes before statistical testing. This method is used in EdgeR for normalization.

### 2.5 Droplet Digital PCR (ddPCR) Validation

MiRNAs identified as significantly expressed across all groups were validated by ddPCR. Reverse transcription and PCR were performed using either One‐Step RT‐ddPCR kit (BIO‐RAD, Hercules, CA, USA) or the TaqMan microRNA reverse transcription kit (Applied Biosystems, Waltham, MA, USA), followed by the ddPCR Supermix for Probes (no dUTP) kit (BIO‐RAD, Hercules, CA, USA). Taqman probes (Applied Byosistems, Waltham, MA, USA) labeled with FAM or VIC dyes were used. Results were analyzed using a Qx100 Droplet Reader instrument (BIO‐RAD, Hercules, CA, USA) and QuantaSoft v.1.7.4 software (BIO‐RAD, Hercules, CA, USA). Data were analyzed in triplicate, and analyses were repeated when reproducibility was less than 10%.

### 2.6 Biological Pathway Analysis

Potential physiological pathways related to the miRNA were identified through a KEGG analysis. KEGG is a database collection covering genomes, biological pathways, diseases, drugs, and chemical substances. The DIANA miRPath tool was used to perform the analysis [29].

### 2.7 Statistical Analysis

Differential miRNA expression analysis was performed by bioNova Científica S.L. (Madrid, Spain), utilizing the “Empirical analysis of DGE” algorithm in CLC Genomics Workbench 21.0.4 with default settings. This method is based on the “Exact Test” for two‐group comparisons developed by [30] and incorporated into the EdgeR Bioconductor package. For validation of study groups via ddPCR, statistical analysis was conducted using R Software version 4.4.1. The Shapiro–Wilk test was used to assess the normality of quantitative variables, and differences between groups were evaluated using the nonparametric Mann–Whitney *U*‐test. A separate analysis excluding patients with spleen removal was also performed. Correlations between bone complications and specific miRNAs were assessed using the Spearman test. Statistical significance was set at *p* < 0.05.

## 3 Results

### 3.1 Patients

A total of 60 untreated patients with GD were included in this study. The patients were proportionally distributed across the three groups, with 20 patients per group. Details regarding gender, median age, the number of splenectomized patients, *GBA1* genotype, and S‐MRI score are provided in Table 1.

**TABLE 1:** Patients' general characteristics.

BD group distribution (*N*)	Mild (MiBD = 20)	Moderate (MoBD = 20)	Severe (SBD = 20)
Female:male	9:11	11:9	9:11
Median age (Q1–Q3)	19.0 (4.00–40.00)	40.5 (28.25–56.00)	37.5 (31.25–47.00)
Splenectomy	1	4	8
*GBA1* genotype			
[c.1226A>G]; [c.1226A>G]	1	3	1
[c.1226A>G]; [c.1448 T>C]	3	7	7
[c.1226A>G]; [**]	11	7	10
[**]; [**]	5	3	2
S‐MRI score	0.0 (0.00–3.75)	9.0 (7.00–10.00)	15.0 (14.00–20.00)

*Note: GBA1* reference sequence: NM_000157. [**]: other pathogenic variant. S‐MRI score is represented as median (Q1–Q3).

Abbreviation: BD: bone disease.

**FIGURE 1:**
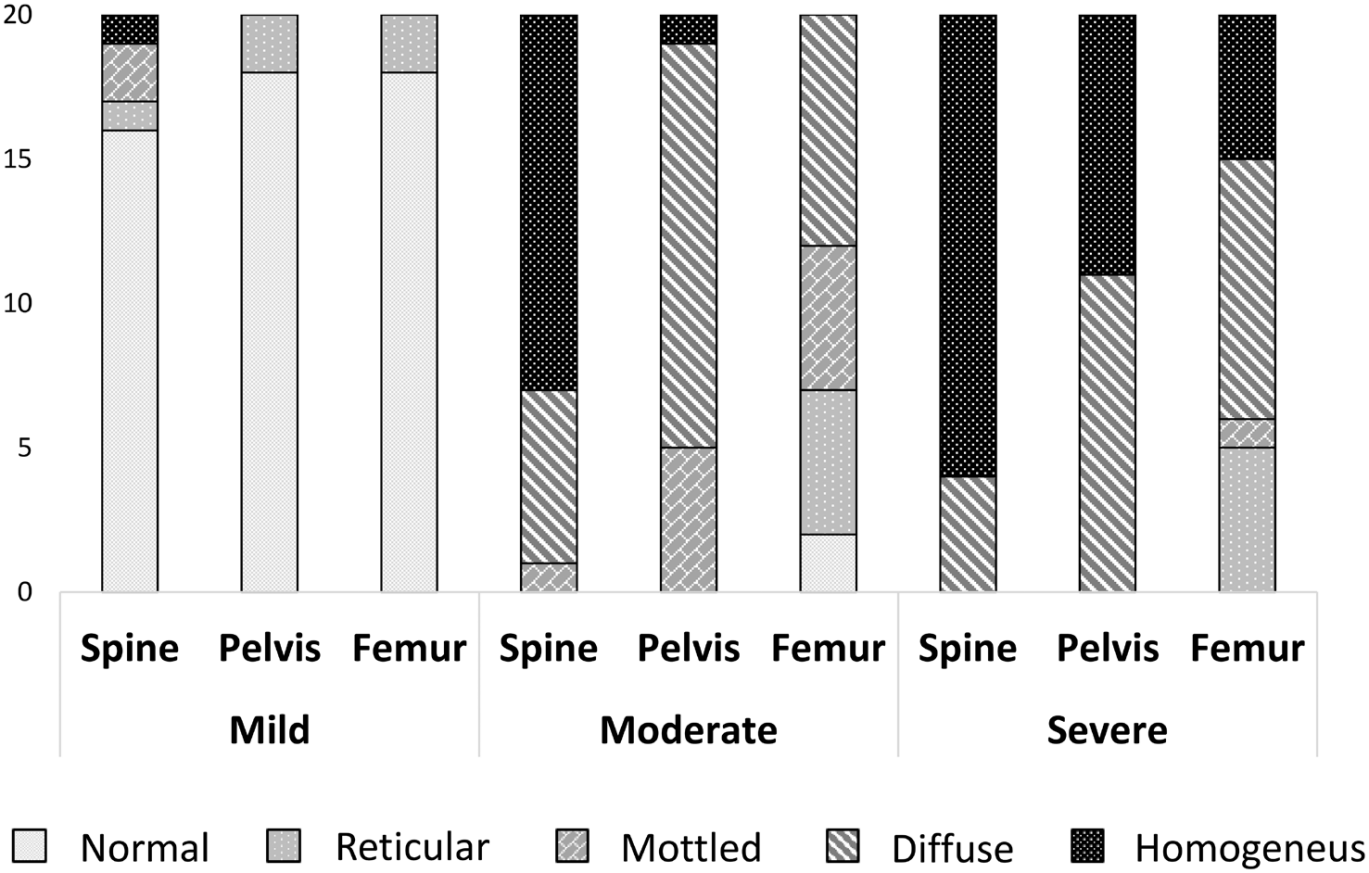
MRI pattern in patients with mild, moderate, and severe bone disease.

In terms of bone involvement, 2 out of 20 patients in the MiBD group and 2 out of 20 in the MoBD group experienced bone crisis. In contrast, the percentage of patients with bone crises doubled in the SBD group, with 4 out of 20 affected. No cases of avascular necrosis or infarcts were observed in the MiBD or MoBD groups. However, in the SBD group, these complications occurred in 7 out of 20 patients and 15 out of 20 patients, respectively. Figure 1 illustrates the MRI patterns observed in the spine, pelvis, and femur for each group.

### 3.2 Exosomes Characterization

**FIGURE 2:**
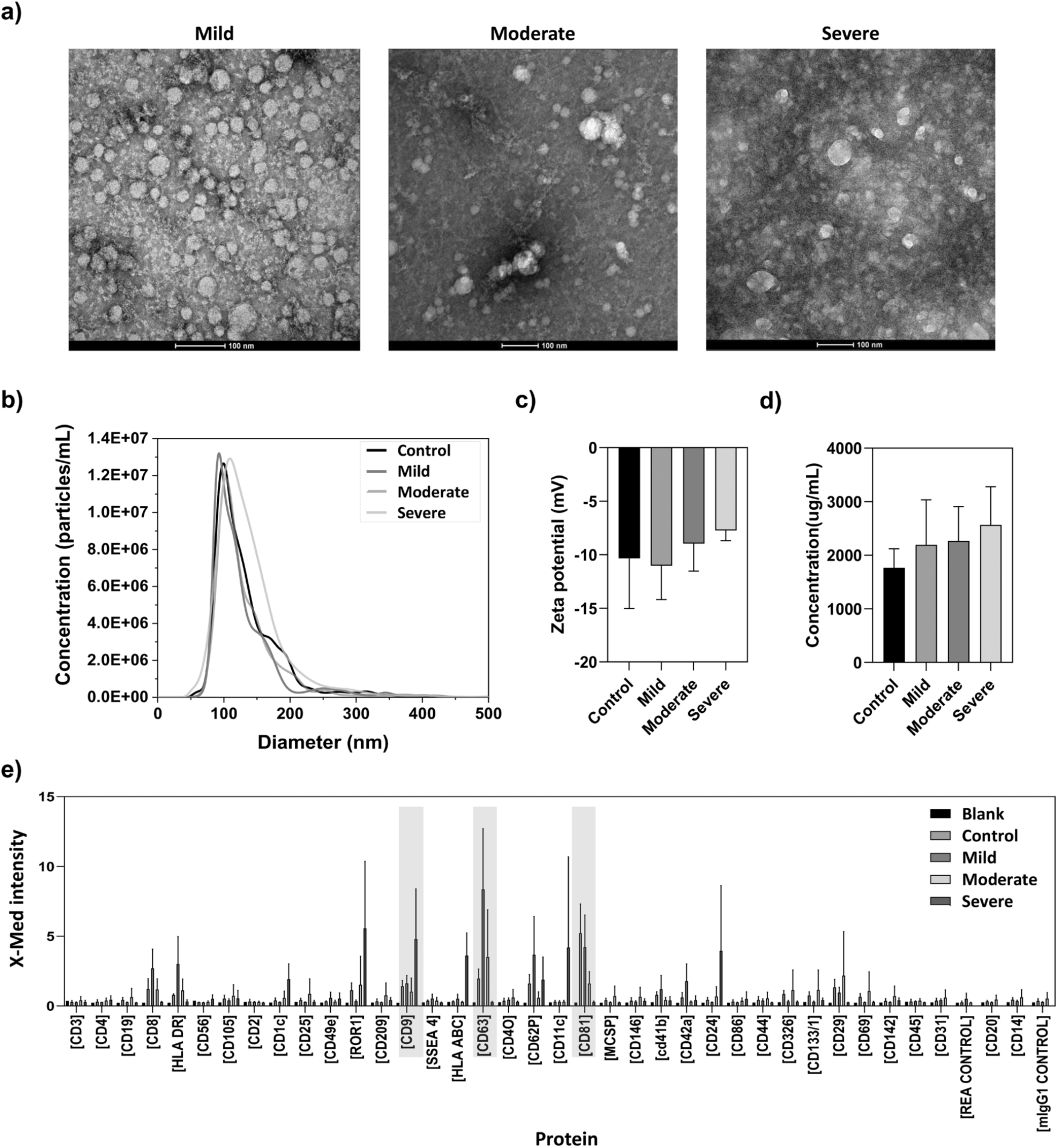
Characterization of exosomes from patients with GD. (a) TEM images of exosomes isolated from patients' plasma (scale bar: 100 nm). (b) NTA analysis of exosomes from healthy donors and mild, moderate, and severe patients with GD. (c) Surface charge analysis of isolated exosomes in terms of zeta potential. (d) BCA analysis of total protein content across groups. (e) MACSPlex analysis of exosomal protein by flow cytometry.

As expected, TEM revealed round‐shaped vesicles with an average diameter ranging from 30 to 150 nm (Figure 2a). NTA data shown in Figure 2b indicate a mean diameter of approximately 130 nm, consistent with the TEM images. Moreover, no significant differences in exosome diameter were observed between the groups (healthy donors, MiBD, MoBD, and SBD patients with GD). The surface charge of exosomes, measured at pH 7, was −10.3 ± 4.7 mV, −11.0 ± 3.1 mV, −8.9 ± 2.6 mV, and −7.7 ± 1.0 mV for healthy controls, MiBD, MoBD, and SBD groups, respectively (Figure 2c).

The comparison of total protein amounts of exosomes, as determined by BCA analysis (Figure 2d), showed no statistically significant differences, although a trend was observed: patients with more severe bone disease appeared to secrete a higher amount of exosomes. Despite the lack of statistical significance, our data suggest that patients with GD (even those in the mild group) tend to secrete higher amounts of exosomes, as reflected by the total protein content, compared to healthy donors. To confirm the exosomal origin of the isolated EVs, we analyzed the expression of exosomal markers by flow cytometry. All samples exhibited high levels of tetraspanins (CD63, CD9, and CD81), which are overexpressed in the exosomal biogenesis pathway, confirming that the isolated vesicles were exosomes and no other types of secreted vesicles (Figure 2e).

### 3.3 Differential Expression of miRNAs

Differential expression patterns of miRNAs were observed between the MoBD and SBD groups compared to the MiBD group. No significant differences were detected between the MoBD and SBD groups. Twelve miRNAs showed significant differences in expression between groups when the data were corrected for age and sex (Table 2).

**TABLE 2:** Significant miRNAs distribution according to bone severity degree.

miRNA	Correction by age		Correction by sex	
	Fold change	*p*	Fold change	*p*
Moderate vs. mild group				
hsa‐miR‐184	22.56	1.4e−23	20.52	2.6e−30
hsa‐miR‐197‐3p	−2.17	0.000036	−2.22	0.0000014
hsa‐miR‐127‐3p	−2.52	0.000028	−2.78	1.6e−7
hsa‐miR‐3615	−1.87	0.000041	−1.78	0.000015
Severe vs. mild group				
hsa‐miR‐184	37.12	1.2e−34	36.48	4.1e−43
hsa‐miR‐206	25.08	3.5e−13	10.70	1.6e−9
hsa‐miR‐12 136	3.12	0.0000083	3.55	1.9e−7
hsa‐miR‐1‐3p	2.96	0.00020	2.99	0.000030
hsa‐miR‐148a‐3p	1.87	0.000047	1.82	0.000024
hsa‐miR‐3613‐5p	−2.32	0.0000029	−2.22	0.0000015
hsa‐miR‐4433b‐5p	−2.80	0.00022	−2.78	0.000055
hsa‐miR‐660‐5p	−1.95	0.000032	−2.18	1.4e−7
hsa‐miR‐92a‐3p	−1.64	0.000013	−1.55	0.000016

*Note:* The “Empirical analysis of DGE” algorithm of the CLC Genomics Workbench 21.0.4 was used for differential expression analysis.

One miRNA, hsa‐miR‐184, was significantly differentially expressed in both the MoBD and SBD groups compared to the MiBD group. The highest fold change in hsa‐miR‐184 expression was observed in the SBD group (37.12 and 36.48, corrected for age and sex, respectively), while the lowest concentration was detected in the MoBD group (22.56 and 20.52, corrected for age and sex, respectively). (Table 2).

### 3.4 ddPCR Validation

**FIGURE 3:**
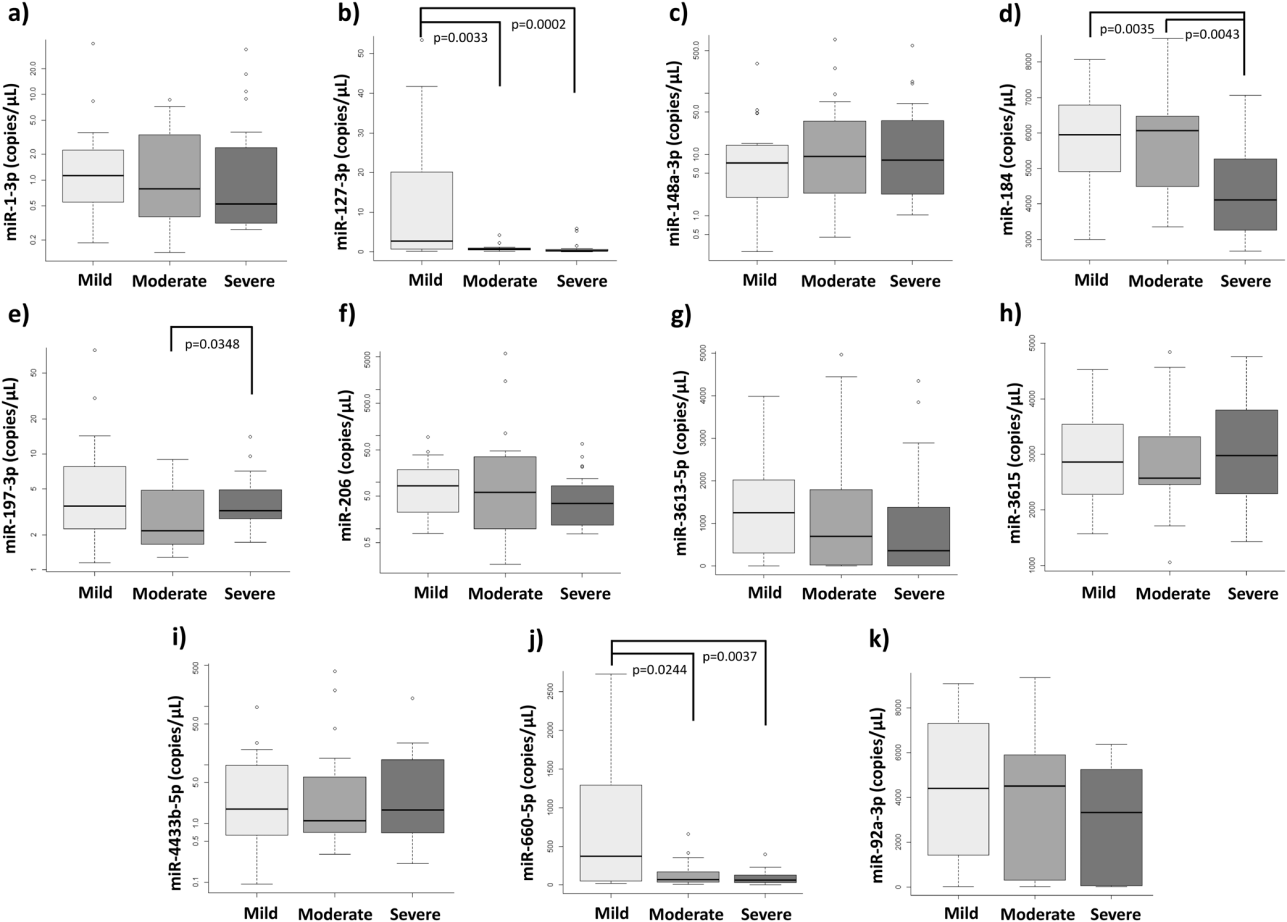
Differential miRNA expression validated by ddPCR. (a) hsa‐miR‐1‐3p. (b) hsa‐miR‐127‐3p. (c) hsa‐miR‐148a‐3p. (d) hsa‐miR‐184. (e) hsa‐miR‐197‐3p. (f) hsa‐miR‐206. (g) hsa‐miR‐3613‐5p. (h) hsa‐miR‐3615. (i) hsa‐miR‐4433b‐5p. (j) hsa‐miR‐660‐5p. (k) hsa‐miR‐92a‐3p.

We validated the differentially expressed miRNAs, and statistically significant differences were observed in the expression levels of four miRNAs: hsa‐miR‐127‐3p, hsa‐miR‐184, hsa‐miR‐197‐3p, and hsa‐miR‐660‐5p (Figure 3). A significantly lower expression of hsa‐miR‐127‐3p was found in both the MoBD (*p* = 0.0033) and SBD (*p* = 0.0002) groups compared to the MiBD group. A similar decrease in expression was observed for hsa‐miR‐660‐5p when comparing the MiBD group with the MoBD (*p* = 0.0244) and the SBD (*p* = 0.0037) groups. In the case of hsa‐miR‐184, significantly lower levels were found in the SBD group compared to both the MiBD (*p* = 0.0035) and MoBD (*p* = 0.0043) groups. Finally, hsa‐miR‐197‐3p showed significantly higher expression in the SBD group (*p* = 0.0348) compared to the MoBD group.

**FIGURE 4:**
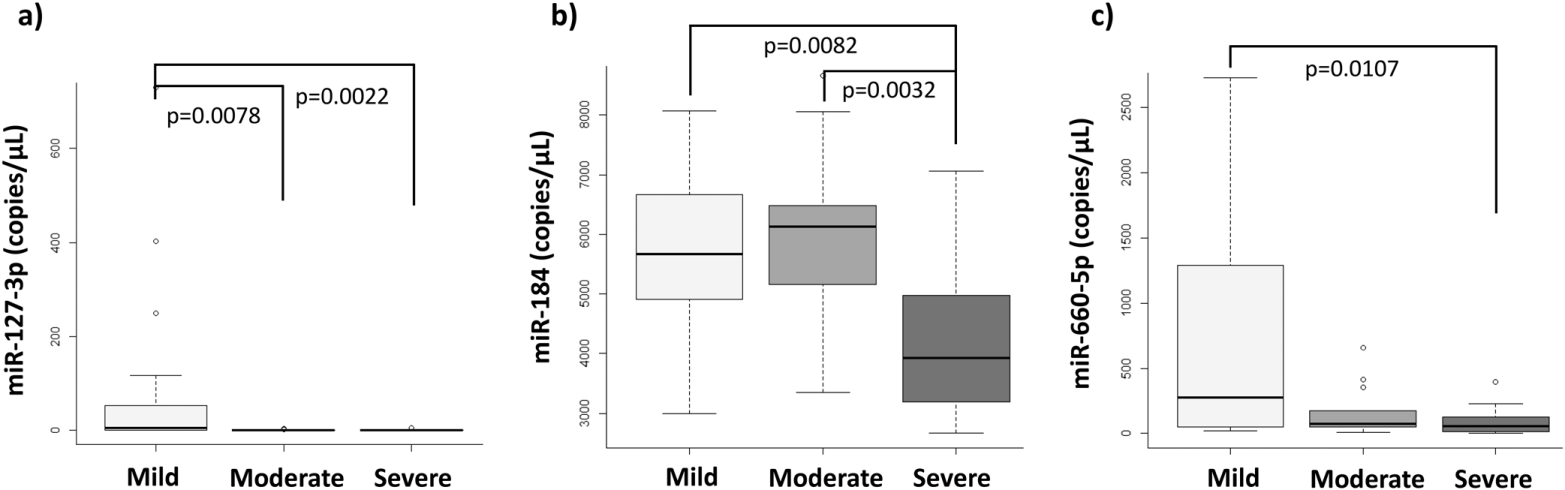
Differential miRNA expression validated by ddPCR after excluding splenectomized patients. (a) hsa‐miR‐127‐3p. (b) hsa‐miR‐184. (c) hsa‐miR‐660‐5p.

Given the influence of splenectomy on the severity of bone involvement in patients with GD, we investigated its effect on the differentially expressed miRNAs obtained from NGS. First, we compared splenectomized (*N* = 13) and non‐splenectomized patients (*N* = 47), and no significant differences were observed in any of the 11 miRNAs studied. Next, we analyzed differences between the groups (MiBD, MoBD, and SBD), excluding splenectomized patients from each group. The results were similar to those obtained when splenectomized patients were included, with one exception: no significant differences in hsa‐miR‐197‐3p expression were found when splenectomized patients were excluded (Figure 4). A significantly lower expression of hsa‐miR‐127‐3p was observed in the MoBD (*p* = 0.0078) and SBD (*p* = 0.0022) groups compared to the MiBD group (Figure 4a). Significantly lower levels of hsa‐miR‐184 were found in the SBD group compared to the MiBD (*p* = 0.0082) and MoBD (*p* = 0.0032) groups (Figure 4b). Additionally, a significantly lower expression of hsa‐miR‐660‐5p was observed in the SBD (*p* = 0.0107) group compared to the MiBD group (Figure 4c).

### 3.5 Biological Pathway Analysis

Physiological pathways influenced by the four miRNAs were explored, leading to the identification of 12 experimentally validated pathways and 13 associated genes in the KEGG database (Table 3). Among these, osteoclast differentiation emerged as one of the most significant pathways.

**TABLE 3:** KEGG pathways for the differentially expressed miRNA as determined by ddPCR.

Rank	KEGG pathway	Genes	*p*
1	Osteoclast differentiation	*IL1R1*, *CYLD*, *AKT2*, *NFATC2*	0.0013
2	Non‐small cell lung cancer	*AKT2*, *FOXO3*	0.0013
3	B cell receptor signaling pathway	*INPPL1*, *AKT2*, *NFATC2*	0.0020
4	Apoptosis	*IL1R1*, *AKT2*, *PRKAR2A*	0.0088
5	Pancreatic cancer	*RAD51*, *AKT2*	0.0176
6	Insulin signaling pathway	*INPPL1*, *AKT2*, *PRKAR2A*	0.0180
7	Endometrial cancer	*AKT2*, *FOXO3*	0.0180
8	VEGF signaling pathway	*AKT2*, *NFATC2*	0.0305
9	HTLV‐1 infection	*IL1R1*, *XBP1*, *AKT2*, *NFATC2*	0.0341
10	Inositol phosphate metabolism	*INPPL1*, *ISYNA1*	0.0402
11	Peroxisome	*PIPOX*, *PEX13*	0.0417
12	Hepatitis B	*ATF6B*, *AKT2*, *NFATC2*	0.0417

### 3.6 Correlation Between miRNA Expression and Bone Complications

A strong correlation was observed between hsa‐miR‐127‐3p and hsa‐miR‐660‐5p (*ρ* = 0.88; *p* = 2.2e^−16^). Additionally, hsa‐miR‐197‐3p displayed a weaker correlation with hsa‐miR‐127‐3p and hsa‐miR‐660‐5p (*ρ* = 0.28; *p* = 0.04 and *ρ* = 0.34; *p* = 0.01, respectively). In contrast, hsa‐miR‐184 did not show any correlation with the other three miRNAs.

When analyzing the relationship between miRNAs expression and bone complications (Table 4), it was found that lower levels of hsa‐miR‐127‐3p, hsa‐miR‐660‐5p, and hsa‐miR‐184 were correlated with the presence of infarcts, necrosis, and the degree of infiltration in the spine, pelvis, and femur.

**TABLE 4:** Correlation between miRNA expression and bone complications.

Complications	hsa‐miR‐127‐3p		hsa‐miR‐184		hsa‐miR‐197‐3p		hsa‐miR‐660‐5p	
	*p*	*ρ*	*p*	*ρ*	*p*	*ρ*	*p*	*ρ*
Bone crisis	0.962	0.007	0.896	−0.017	0.966	−0.006	0.564	0.080
Infarct	0.003	−0.402	0.030	−0.283	0.468	0.096	0.031	−0.293
Necrosis	0.001	−0.435	0.011	−0.329	0.516	−0.085	0.040	−0.280
Spine infiltration	0.004	−0.390	0.029	−0.285	0.680	−0.054	0.039	−0.281
Pelvis infiltration	0.00002	−0.548	0.001	−0.407	0.863	0.023	0.002	−0.409
Femur infiltration	0.0001	−0.500	0.005	−0.361	0.973	−0.004	0.009	−0.353

## 4 Discussion

The role of miRNAs in gene regulation is well established, and there is a growing body of research investigating their involvement in various diseases. These molecules not only provide insights into disease pathophysiology but also hold promise as diagnostic biomarkers and therapeutic targets. In the context of GD, research on miRNAs remains limited. The first published study identified several miRNAs, including miR‐127‐5p, miR‐16‐5p, and miR‐195‐5p, as modulators of β‐glucocerebrosidase (GCase) activity [16]. In our study, we did not find a correlation between miR‐127‐5p and bone disease; however, its complementary sequence (miR‐127‐3p) was significantly decreased in more severe forms of GD, potentially affecting the same regulatory mechanism (GCase transporter).

Other studies have focused on the relationship between miRNAs and the neuronopathic form of GD, reporting that a miRNA dysregulation is linked to neuronal guidance, synaptic plasticity, and inflammation [17, 19]. A recent study from 2021 was conducted on 20 adult patients with GD1 undergoing ERT, comparing miRNA profiles to those in the healthy population [18]. That study identified miR‐26b‐5p as significantly elevated in GD patients, with an association to the *TRPS1* gene, which is involved in the regulation of bone and cartilage growth [18]. In our study, miR‐26b‐5p was not significantly expressed, which could be due to differences in study design.

Bone disease was not a primary focus in the study by Pawliński et al., and patients were not classified based on this characteristic. Additionally, their cohort included a mix of patients with and without GD‐modifying treatments. Another key distinction lies in the source of miRNAs: while their study analyzed plasma‐derived miRNAs, our study focuses on exosomal miRNAs, ensuring that the analyzed content more accurately reflects the metabolic state of the parental cells [31]. We also confirmed the exosomal origin of miRNAs using multiple physicochemical and biological techniques, including size and morphology analysis. Consistent with previous publications, the diameters measured by NTA were slightly larger than those observed by TEM [32, 33]. Furthermore, we characterized the negatively charged surface of the exosomes—attributed to the presence of negatively charged phospholipids and proteins on the exosomal membrane [33] as well as the expression of exosome‐associated proteins on their membrane.

When assessing the total amount of secreted exosomes, no statistically significant differences were observed; however, a clear trend emerged: patients with more severe bone disease in GD appeared to secrete higher amounts of exosomes. This suggests that as bone disease severity increases in GD, exosome secretion also rises. Recent studies have shown that impaired lysosomal function leads to an accumulation of cellular waste, prompting the cell to increase vesicle export as a compensatory mechanism [34, 35, 36, 37]. In GD, it has been reported that an increase in exosome secretion correlates with decreased GCase activity [26]. Additionally, a clinical trial (NCT05843552) is currently investigating EVs as potential biomarkers and therapeutic targets, highlighting their possible clinical applications [38]. As far as we know, this is the first study to describe miRNAs isolated from exosomes derived from patients with GD. With further research and larger patient cohorts, these miRNAs could become valuable tools for monitoring and predicting bone involvement in GD.

The results regarding the exosomal miRNAs that were found to be altered between groups demonstrated that the downregulation of hsa‐miR‐127‐3p, hsa‐miR‐184, and hsa‐miR‐660‐5p is associated with the severity of bone involvement. In our study, we observed discrepancies between NGS and ddPCR results for certain miRNAs, such as miR‐184. These differences are not uncommon and arise from the intrinsic characteristics of each method. NGS was used as a high‐throughput screening tool to identify potentially differentially expressed miRNAs, but it is not designed for precise quantification and can be affected by amplification biases, platform‐specific effects, and normalization challenges [39, 40, 41]. In contrast, ddPCR enables absolute quantification with high precision and reproducibility, making it the preferred method for validating and quantifying specific miRNAs. In our case, ddPCR data were analyzed in triplicate, showing high consistency across replicates. Therefore, although NGS and ddPCR yielded opposite results for miR‐184, we believe this discrepancy reflects methodological differences between the platforms rather than a technical error or lack of biological relevance. Overall, we consider the ddPCR results to be more reliable for accurate quantification of selected miRNAs.

A review of the affected physiological pathways revealed that osteoclast differentiation was the most significant (*p* = 0.0013), involving the *IL1R1*, *CYLD*, *AKT2*, and *NFATC2* genes. These genes are related to osteoclast function and bone resorption [42, 43, 44, 45]. However, no existing literature has linked these genes specifically to GD or its bone involvement, despite the well‐established relationship between osteoclast differentiation and GD [46, 47]. What has been reported so far is the relationship between different molecules related to interleukin 1 (IL‐1) and bone involvement in GD. One study indicates that a haplotype found in IL‐1 genes (*IL1α*, *IL1β*, and *IL1RN*) in patients with GD correlates with the loss of bone mineral density [48]. Another study suggests that the IL1R2 protein could serve as a promising biomarker to predict the early diagnosis of skeletal complications in GD1 patients [11]. These findings highlight the importance of bone remodeling processes, which would have been expected if patients had been selected based on bone mineral density loss. However, in our study, patients were selected based on intraosseous vascular complications and the degree of bone infiltration.

Patients included in this study were selected using the S‐MRI score, which includes different bone alterations, such as bone marrow infiltration, bone crises, avascular necrosis, and bone infarcts. It was observed that hsa‐miR‐127‐3p, hsa‐miR‐660‐5p, and hsa‐miR‐184 were correlated with the presence of infarcts, necrosis, and the degree of infiltration in the spine, pelvis, and femur. The strong correlation of hsa‐miR‐127‐3p, hsa‐miR‐660‐5p, and hsa‐miR‐184 with the degree of infiltration in the femur is of special relevance because it is one of the most serious indicators for the bone disease [49]. Hsa‐miR‐197‐3p was not found to correlate with any of the bone‐related complications studied. Furthermore, when splenectomized patients were excluded from the group analysis, no significant differences were observed for this miRNA. For the reasons mentioned above, we propose that the other three miRNAs (hsa‐miR‐127‐3p, hsa‐miR‐184, and hsa‐miR‐660‐5p) could be considered as potential biomarkers for bone involvement in GD.

Reinforcing the results linking miRNAs to different bone complications, this study is not the first to associate the downregulation of miR‐127‐3p with osteonecrosis. A 2019 study by Hong et al. demonstrated that miR‐127‐3p is downregulated in serum and bone samples from patients with alcohol‐induced osteonecrosis of the femoral head, compared to control individuals [50]. Although there is no direct evidence linking miRNAs to bone infarcts, other studies have shown that the overexpression of hsa‐miR‐184 and hsa‐miR‐127‐3p can influence ischemic damage in the brain and heart by affecting cell survival, inflammation, and apoptosis pathways [51, 52]. While both conditions share a common physiological mechanism, they differ in the specific areas of bone affected.

The correct structure of the inner bone is altered in several diseases, including GD, where bone infiltration plays an important role. However, other factors may contribute to bone complications, and hsa‐miR‐660‐5p downregulation has been associated with changes in inner bone structure in other diseases, such as acromegaly. In acromegaly, the impact of this miRNA is strongly positively linked to osteoprotegerin levels, and since it is under expressed, an increase in the osteoclastogenic process would be expected in these patients [53]. The same pathway can be affected in Gaucher patients, due to the commonality in the hsa‐miR‐660‐5p reduction between both diseases.

As previously mentioned, bone marrow infiltration is a crucial factor in bone involvement in GD, present in nearly all patients. While none of these miRNAs have been specifically linked to macrophage bone infiltration, hsa‐miR‐127‐3p has been shown to play an important role in macrophage polarization and activity [54]. Hsa‐miR‐127‐3p enhances inflammatory signaling pathways, which could contribute to increased macrophage activity and tissue infiltration [54]. The observed downregulation of hsa‐miR‐127‐3p in patients with GD, despite its role in inflammation and tissue infiltration, could be explained by compensatory pathways that modulate its expression in response to the heightened inflammatory microenvironment in GD. Regarding hsa‐miR‐184, this miRNA is not directly related to bone marrow infiltration, but it has been shown to play various roles in bone marrow‐related processes, particularly in bone marrow stromal cells (BMSCs). In one study, hsa‐miR‐184 was involved in regulating BMSCs in osteoporosis models by influencing the expression of specific proteins important for osteogenic differentiation [55].

In summary, we identified three possible candidates (hsa‐miR‐127‐3p, hsa‐miR‐184, and hsa‐miR‐660‐5p) as potential biomarkers for bone disease severity in GD. These findings may enhance our understanding of the molecular mechanisms underlying the different manifestations of GD and help us predict their occurrence, especially for irreversible conditions such as osteonecrosis and bone infarcts. Future studies could expand on these results to assess the consistency and reproducibility of our findings. Additionally, examining patients undergoing different treatments may provide insights into how these therapies impact miRNA expression and the progression of bone disease.

## 5 Conclusions

This study is the first to isolate miRNAs from exosomes derived from patients with GD and associate their expression with bone involvement severity. We found that exosomal hsa‐miR‐127‐3p, hsa‐miR‐184, and hsa‐miR‐660‐5p expression is significantly reduced in patients with more severe bone disease. These miRNAs also correlate with different bone manifestations. Our results suggest that these miRNAs, and in particular the combination of hsa‐miR‐127‐3p or hsa‐miR‐660‐5p with hsa‐miR‐184, could serve as accessible and reliable biomarkers for assessing bone involvement in GD. Additionally, these findings provide valuable insights into the pathophysiology of bone disease in GD and may inform the development of therapeutic strategies targeting miRNA mechanisms. However, further investigation and revalidation with a larger number of patients would be necessary.

## Author Contributions

Irene Serrano‐Gonzalo, Laura López de Frutos, and Pilar Giraldo contributed to the study design and data interpretation. Irene Serrano‐Gonzalo, Maria Sancho‐Albero, and Mercedes Roca‐Espiau contributed to the data analysis. Irene Serrano‐Gonzalo, Laura López de Frutos, Ralf Köhler, and Pilar Giraldo contributed to the acquisition and interpretation of data. All authors had full access to the data, participated fully in drafting and revising the manuscript, and approved the final version of the manuscript. All authors have agreed both to be accountable for all aspects of the work and to ensuring that questions related to the accuracy or integrity of any part of the work are appropriately investigated and resolved.

## Ethics Statement

This study was performed in line with the principles of the Declaration of Helsinki. This study was approved by the Research Ethics Committee of the Community of Aragon (PI20/264).

## Consent

All procedures followed were in accordance with the ethical standards of the responsible committee on human experimentation (institutional and national) and with the Helsinki Declaration of 1975, as revised in 2000. Informed consent was obtained from all patients for being included in the study.

## Conflicts of Interest

Irene Serrano‐Gonzalo has received support from Sanofi, Takeda, Chiesi, and Alexion to attend scientific meetings and conferences. Laura López de Frutos is an employee of Azafaros B.V. outside the submitted work without stock options. Pilar Giraldo received research grants from Sanofi‐Genzyme, Takeda, Alexion, and Pfizer; all of them have been deposited into the Spanish Foundation for the Study and Treatment of Gaucher Disease (FEETEG) to contribute to the development of research in lysosomal storage disorders. The remaining authors declare no conflicts of interest.

## Data Availability

The datasets generated during the current study are not publicly available but are available from the corresponding author on reasonable request.
